# Crystal structure of {[2-hy­droxy-2-(3-meth­oxy­phen­yl)cyclo­hex­yl]meth­yl}di­methyl­ammonium benzoate

**DOI:** 10.1107/S2056989015019362

**Published:** 2015-10-17

**Authors:** S. N. Sheshadri, P. Nagendra, B. P. Siddaraju, K. H. Hemakumar, K. Byrappa, N. K. Lokanath, S. Madan Kumar

**Affiliations:** a Department of Chemistry, GSSS Institute of Engineering Technology for Women, Mysuru 570 016, India; b Department of Chemistry, BET Academy of Higher Education, Bharathi College, Bharthi Nagara, Mandya 571 422, India; c Department of Engineering Chemistry, Cauvery Institute of Technology, Mandya 571 402, India; d Department of Chemistry, Cambridge institute of Technology, Bengaluru 560 036, India; e Department of Materials Science, Mangalagangotri, Mangalore University, Mangaluru 574 199, India; f Department of Studies in Physics, University of Mysore, Manasagangotri, Mysore, 570 006, India; g PURSE Lab, Mangalagangotri, Mangalore University, Mangaluru 574 199, India

**Keywords:** crystal structure, Tramadol, inter­molecular hydrogen bonds

## Abstract

The title compound, C_16_H_26_NO_2_^+^·C_7_H_5_O_2_^−^, is a benzoate salt of the painkiller Tramadol. The six-membered cyclo­hexane ring of the cation adopts a slightly distorted chair conformation and carries OH and 3-meth­oxy­phenyl substituents at the 2-position and a protonated methyl­aza­niumylmethyl group at the 3-position. In addition, a weak intra­molecular C—H⋯O hydrogen bond is observed in the cation. In the crystal, weak O—H⋯O, N—H⋯O and C—H⋯O hydrogen bonds link the components into chains along [010]. A C—H⋯π contact is also observed.

## Related literature   

For pharmaceutical applications of Tramadol and related analgesics, see: Scott & Perry (2000 ▸). For related structures, see: Tessler & Goldberg (2004 ▸); Arman *et al.* (2010 ▸); Hemamalini & Fun (2010 ▸); Siddaraju *et al.* (2011 ▸); Lin & Zhang (2013 ▸); Smith (2014 ▸); Jasinski *et al.* (2015 ▸); Sun *et al.* (2012 ▸).
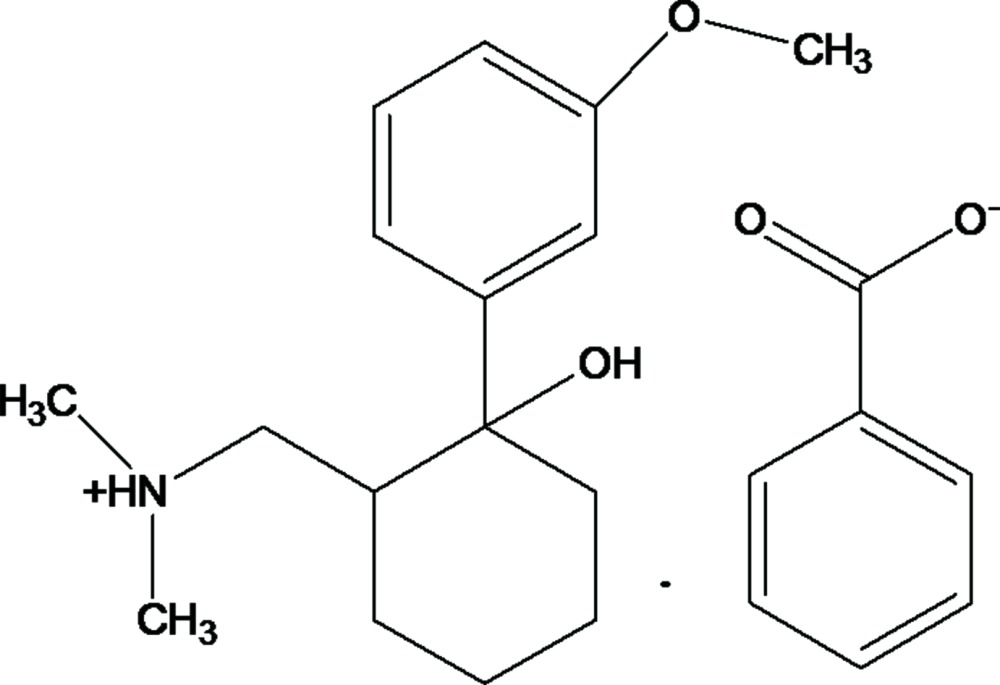


## Experimental   

### Crystal data  

C_16_H_26_NO_2_^+^·C_7_H_5_O_2_^−^
*M* *_r_* = 385.49
Triclinic, 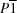

*a* = 9.013 (4) Å
*b* = 9.767 (4) Å
*c* = 12.726 (6) Å
α = 75.008 (16)°
β = 89.79 (2)°
γ = 76.493 (16)°
*V* = 1050.3 (8) Å^3^
*Z* = 2
Mo *K*α radiation
μ = 0.08 mm^−1^
*T* = 293 K
0.55 × 0.51 × 0.3 mm

### Data collection  

Rigaku Saturn724+ diffractometer
Absorption correction: multi-scan (*NUMABS*; Rigaku 1999 ▸) *T*_min_ = 0.955, *T*_max_ = 0.975
11931 measured reflections
5660 independent reflections
3504 reflections with *I* > 2σ(*I*)
*R*_int_ = 0.029

### Refinement  

*R*[*F* ^2^ > 2σ(*F* ^2^)] = 0.053
*wR*(*F* ^2^) = 0.134
*S* = 1.03
5660 reflections
257 parameters
H-atom parameters constrained
Δρ_max_ = 0.17 e Å^−3^
Δρ_min_ = −0.21 e Å^−3^

### 

Data collection: *CrystalClear SM Expert* (Rigaku, 2011 ▸); cell refinement: *CrystalClear SM Expert*; data reduction: *CrystalClear SM Expert*; program(s) used to solve structure: *SHELXS97* (Sheldrick, 2008 ▸); program(s) used to refine structure: *SHELXL2014* (Sheldrick, 2015 ▸); molecular graphics: *OLEX2* (Dolomanov *et al.*, 2009 ▸) and *Mercury* (Macrae *et al.*, 2008 ▸); software used to prepare material for publication: *OLEX2*.

## Supplementary Material

Crystal structure: contains datablock(s) I. DOI: 10.1107/S2056989015019362/sj5480sup1.cif

Structure factors: contains datablock(s) I. DOI: 10.1107/S2056989015019362/sj5480Isup2.hkl

Supporting information file. DOI: 10.1107/S2056989015019362/sj5480Isup3.cml

**Fig. 1.**
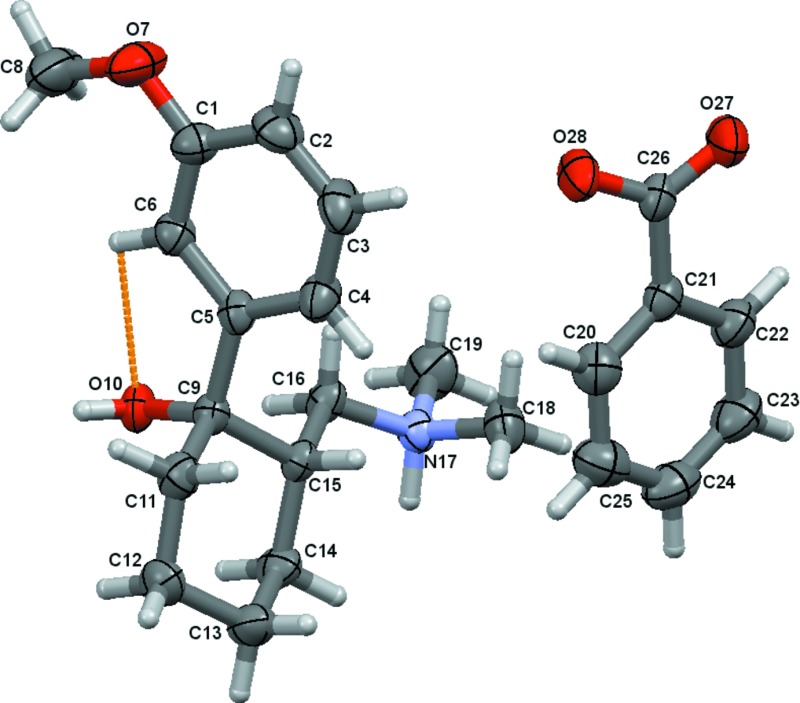
A view of the title molecule, with atom labelling. Displacement ellipsoids are drawn at the 50% probability level and an intramolecular hydrogen bond is drawn as a dashed line.

**Fig. 2.**
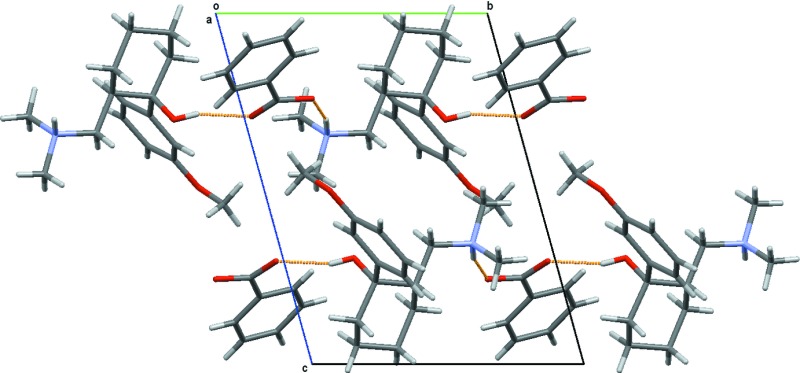
A viewed along the a axis of the crystal packing of the title compound. Hydrogen bonds are drawn as a dashed lines.

. DOI: 10.1107/S2056989015019362/sj5480fig1.tif
A view of the title mol­ecule, with atom labelling. Displacement ellipsoids are drawn at the 50% probability level and an intra­molecular hydrogen bond is drawn as a dashed line.

a . DOI: 10.1107/S2056989015019362/sj5480fig2.tif
A viewed along the *a* axis of the crystal packing of the title compound. Hydrogen bonds are drawn as a dashed lines.

CCDC reference: 1425991

Additional supporting information:  crystallographic information; 3D view; checkCIF report

## Figures and Tables

**Table 1:** Hydrogen-bond geometry (, ) *Cg*1 is the centroid of the C1C6 benzene ring.

*D*H*A*	*D*H	H*A*	*D* *A*	*D*H*A*
C6H6O10	0.93	2.42	2.775(2)	102
C14H14*B*O28^i^	0.97	2.41	3.251(2)	144
N17H17O28^i^	0.98	1.65	2.6120(18)	165
O10H10O27^ii^	0.82	1.94	2.7269(17)	161
C23H23*Cg*1^iii^	0.93	2.83	3.684(3)	153

Symmetry codes: (i) 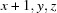; (ii) 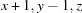; (iii) 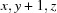.

## supplementary crystallographic information

### S1. Comment

2-((dimethylamino)methyl)-1-(3-methoxyphenyl)cyclohexanol hydrochloride (Tramadol) is used in the treatment of disorders of the central nervous system and in treating extreme pain. This molecule is analogous to the phenanthrene alkaloid codeine and is used in the control of pre-operative pain (Scott & Perry, 2000).

There are number of crystal structures reported available related to this salt. These include Venlafaxine [(*RS*)-1-[2-dimethylamino-1-(4-methoxyphenyl)-ethyl]- cyclohexanol] (Tessler & Goldberg, 2004), benzoic acid-2-{(*E*)-[(*E*)-2-(2-pyridylmethylidene) hydrazin-1-ylidene]methyl}pyridine (2/1) (Arman *et al.*, 2010), 2,3-diaminopyridinium benzoate benzoic acid solvate (Hemamalini & Fun, 2010), Tramadol hydrochloride-benzoic acid (1/1) (Siddaraju *et al.*, 2011), 4-(*cyclo*-propane-carboxamido) benzoic acid (Sun *et al.*, 2012), 3,5-bis[(pyridin-4-yl)meth-oxy]benzoic acid (Lin & Zhang, 2013). The hydrogen-bonded two- and three-dimensional polymeric structures of the ammonium salts of 3,5-di-nitro-benzoic acid, 4-nitro-benzoic acid and 2,4-di-chloro-benzoic acid (Smith, 2014) and 4-[1-(2-hydroxypropyl)-4,5-diphenyl-1*H*-imidazol-2-yl]benzoic acid (Jasinski *et al.*, 2015) have also been reported. In the view of the importance of Tramadol, we report herein the crystal structure of the title compound 2-hydroxy-2-(3-methoxy-phenyl)-cyclohexylmethyl-dimethyl-ammonium benzoate.

In the title molecule (Fig. 1), the six membered cyclohexane ring (C9/C11–C15) adopts a slightly distorted chair conformation with ring puckering parameters Q, θ and φ of 0.5605 (16) Å, 5.06 (16)°, and 210.3 (6)°, respectively. An intramolecular C6—H6···O10 hydrogen bond (Fig 1, Table 1) is found in the cation. Bond lengths are within normal ranges.

The crystal structure is stabilized with weak intermolecular O—H···O, N—H···O and C—H···O hydrogen bonds and a weak intermolecular C23—H23···*Cg1* interaction is also observed Table 1, Fig. 2.

### S2. Experimental

Tramadol (3 g, 0.01 mol) and benzoic acid (1 g, 0.01 mol) were each dissolved in 10 ml of ethanol. The solutions were mixed and stirred in a beaker at 333 K for 30 minutes. The mixture was kept aside for three days at room temperature. Colourless X-ray quality crystals were formed and one was used for the data collection

### S3. Refinement

Crystal data, data collection and structure refinement details are summarized in Table 1. The hydrogen atoms were fixed geometrically (C—H = 0.93–0.96 Å, N—H = 0.98 Å) and allowed to ride on their parent atoms with *U*_iso_(H) = 1.2*U*_eq_(C/N).

### Crystal data

**Table:**

C_16_H_26_NO_2_^+^·C_7_H_5_O_2_^−^	*Z* = 2
*M**_r_* = 385.49	*F*(000) = 416
Triclinic, *P*1	*D*_x_ = 1.219 Mg m^−^^3^
*a* = 9.013 (4) Å	Mo *K*α radiation, λ = 0.71075 Å
*b* = 9.767 (4) Å	Cell parameters from 5660 reflections
*c* = 12.726 (6) Å	θ = 3.0–29.5°
α = 75.008 (16)°	µ = 0.08 mm^−^^1^
β = 89.79 (2)°	*T* = 293 K
γ = 76.493 (16)°	Block, colourless
*V* = 1050.3 (8) Å^3^	0.55 × 0.51 × 0.3 mm

### Data collection

**Table:**

Rigaku Saturn724+ diffractometer	5660 independent reflections
Radiation source: Sealed tube, Rotating Anode	3504 reflections with *I* > 2σ(*I*)
Confocal monochromator	*R*_int_ = 0.029
Detector resolution: 28.5714 pixels mm^-1^	θ_max_ = 29.5°, θ_min_ = 3.0°
profile data from ω–scans	*h* = −12→11
Absorption correction: multi-scan (*NUMABS*; Rigaku 1999)	*k* = −13→13
*T*_min_ = 0.955, *T*_max_ = 0.975	*l* = −17→14
11931 measured reflections	

### Refinement

**Table:**

Refinement on *F*^2^	Primary atom site location: structure-invariant direct methods
Least-squares matrix: full	Hydrogen site location: inferred from neighbouring sites
*R*[*F*^2^ > 2σ(*F*^2^)] = 0.053	H-atom parameters constrained
*wR*(*F*^2^) = 0.134	*w* = 1/[σ^2^(*F*_o_^2^) + (0.0598*P*)^2^] where *P* = (*F*_o_^2^ + 2*F*_c_^2^)/3
*S* = 1.03	(Δ/σ)_max_ = 0.001
5660 reflections	Δρ_max_ = 0.17 e Å^−^^3^
257 parameters	Δρ_min_ = −0.21 e Å^−^^3^
0 restraints	

### Special details

**Table:**

Geometry. All e.s.d.'s (except the e.s.d. in the dihedral angle between two l.s. planes) are estimated using the full covariance matrix. The cell e.s.d.'s are taken into account individually in the estimation of e.s.d.'s in distances, angles and torsion angles; correlations between e.s.d.'s in cell parameters are only used when they are defined by crystal symmetry. An approximate (isotropic) treatment of cell e.s.d.'s is used for estimating e.s.d.'s involving l.s. planes.

### Fractional atomic coordinates and isotropic or equivalent isotropic displacement parameters (Å^2^)

**Table:**

	*x*	*y*	*z*	*U*_iso_*/*U*_eq_	
C1	0.51600 (17)	0.30154 (17)	0.58415 (12)	0.0418 (4)	
C2	0.39216 (17)	0.38352 (18)	0.62190 (13)	0.0502 (4)	
H2	0.2941	0.3959	0.5925	0.060*	
C3	0.41457 (17)	0.44717 (17)	0.70353 (14)	0.0520 (4)	
H3	0.3314	0.5032	0.7290	0.062*	
C4	0.56018 (16)	0.42788 (16)	0.74757 (13)	0.0448 (4)	
H4	0.5738	0.4708	0.8029	0.054*	
C5	0.68653 (15)	0.34553 (14)	0.71072 (11)	0.0332 (3)	
C6	0.66294 (15)	0.28320 (15)	0.62715 (11)	0.0366 (3)	
H6	0.7459	0.2291	0.6001	0.044*	
C8	0.6071 (2)	0.1648 (2)	0.45646 (15)	0.0697 (5)	
H8A	0.5688	0.1311	0.4000	0.104*	
H8B	0.6659	0.0829	0.5111	0.104*	
H8C	0.6707	0.2297	0.4258	0.104*	
C9	0.84766 (14)	0.31719 (13)	0.76345 (10)	0.0310 (3)	
C11	0.85527 (17)	0.21038 (15)	0.87622 (11)	0.0406 (3)	
H11A	0.7688	0.2473	0.9152	0.049*	
H11B	0.8451	0.1174	0.8670	0.049*	
C12	1.00060 (18)	0.18474 (16)	0.94543 (12)	0.0465 (4)	
H12A	1.0865	0.1338	0.9130	0.056*	
H12B	0.9923	0.1239	1.0176	0.056*	
C13	1.0282 (2)	0.32939 (16)	0.95426 (12)	0.0491 (4)	
H13A	0.9471	0.3760	0.9929	0.059*	
H13B	1.1243	0.3118	0.9954	0.059*	
C14	1.03296 (17)	0.42967 (16)	0.84145 (11)	0.0417 (4)	
H14A	1.1171	0.3845	0.8044	0.050*	
H14B	1.0512	0.5210	0.8486	0.050*	
C15	0.88435 (15)	0.46093 (13)	0.77334 (10)	0.0317 (3)	
H15	0.8016	0.5100	0.8107	0.038*	
C16	0.88662 (16)	0.56049 (14)	0.65994 (10)	0.0358 (3)	
H16A	0.9709	0.5144	0.6235	0.043*	
H16B	0.7929	0.5688	0.6190	0.043*	
C18	0.77644 (17)	0.79335 (16)	0.70698 (14)	0.0508 (4)	
H18A	0.6805	0.8004	0.6709	0.076*	
H18B	0.7905	0.8896	0.7005	0.076*	
H18C	0.7767	0.7434	0.7826	0.076*	
C19	0.9145 (2)	0.79324 (17)	0.54203 (12)	0.0572 (5)	
H19A	0.9985	0.7395	0.5112	0.086*	
H19B	0.9315	0.8870	0.5410	0.086*	
H19C	0.8215	0.8062	0.5001	0.086*	
N17	0.90203 (13)	0.71131 (11)	0.65610 (9)	0.0337 (3)	
H17	0.9976	0.7013	0.6970	0.040*	
O7	0.48324 (13)	0.23977 (15)	0.50425 (10)	0.0642 (3)	
O10	0.96103 (10)	0.26098 (10)	0.69699 (8)	0.0372 (2)	
H10	0.9774	0.1717	0.7133	0.056*	
C20	0.38473 (18)	0.75688 (18)	0.88067 (13)	0.0492 (4)	
H20	0.3598	0.6668	0.8964	0.059*	
C21	0.29160 (16)	0.87614 (15)	0.80738 (11)	0.0376 (3)	
C22	0.33245 (18)	1.00897 (17)	0.78461 (12)	0.0471 (4)	
H22	0.2706	1.0903	0.7362	0.056*	
C23	0.4632 (2)	1.0218 (2)	0.83276 (14)	0.0564 (4)	
H23	0.4902	1.1110	0.8155	0.068*	
C24	0.5540 (2)	0.9031 (2)	0.90636 (14)	0.0595 (5)	
H24	0.6417	0.9121	0.9396	0.071*	
C25	0.51482 (19)	0.7706 (2)	0.93080 (13)	0.0597 (5)	
H25	0.5757	0.6903	0.9809	0.072*	
C26	0.15086 (16)	0.86086 (15)	0.75316 (12)	0.0385 (3)	
O27	0.05139 (13)	0.97122 (11)	0.70637 (10)	0.0600 (3)	
O28	0.14406 (12)	0.73144 (11)	0.75774 (9)	0.0529 (3)	

### Atomic displacement parameters (Å^2^)

**Table:**

	*U*^11^	*U*^22^	*U*^33^	*U*^12^	*U*^13^	*U*^23^
C1	0.0387 (8)	0.0477 (9)	0.0414 (8)	−0.0137 (7)	0.0007 (7)	−0.0132 (7)
C2	0.0321 (8)	0.0561 (10)	0.0601 (10)	−0.0087 (7)	−0.0010 (7)	−0.0132 (8)
C3	0.0344 (8)	0.0496 (9)	0.0740 (12)	−0.0053 (7)	0.0125 (8)	−0.0245 (8)
C4	0.0378 (8)	0.0443 (9)	0.0602 (10)	−0.0115 (7)	0.0102 (7)	−0.0262 (7)
C5	0.0323 (7)	0.0281 (7)	0.0405 (8)	−0.0102 (5)	0.0069 (6)	−0.0088 (6)
C6	0.0330 (7)	0.0382 (7)	0.0400 (8)	−0.0092 (6)	0.0056 (6)	−0.0123 (6)
C8	0.0581 (11)	0.0930 (15)	0.0678 (12)	−0.0084 (10)	−0.0036 (9)	−0.0474 (11)
C9	0.0315 (7)	0.0276 (6)	0.0357 (7)	−0.0079 (5)	0.0060 (6)	−0.0110 (5)
C11	0.0485 (9)	0.0320 (7)	0.0422 (8)	−0.0154 (6)	0.0049 (7)	−0.0066 (6)
C12	0.0566 (10)	0.0363 (8)	0.0421 (8)	−0.0103 (7)	−0.0034 (7)	−0.0031 (6)
C13	0.0608 (10)	0.0446 (9)	0.0418 (8)	−0.0145 (8)	−0.0104 (8)	−0.0094 (7)
C14	0.0459 (8)	0.0378 (8)	0.0459 (8)	−0.0181 (7)	−0.0024 (7)	−0.0117 (6)
C15	0.0373 (7)	0.0265 (6)	0.0340 (7)	−0.0107 (5)	0.0048 (6)	−0.0103 (5)
C16	0.0453 (8)	0.0308 (7)	0.0353 (7)	−0.0145 (6)	0.0059 (6)	−0.0109 (6)
C18	0.0457 (9)	0.0358 (8)	0.0688 (11)	−0.0039 (7)	0.0084 (8)	−0.0157 (8)
C19	0.0827 (13)	0.0446 (9)	0.0448 (9)	−0.0267 (9)	0.0087 (9)	−0.0026 (7)
N17	0.0359 (6)	0.0283 (6)	0.0367 (6)	−0.0095 (5)	0.0029 (5)	−0.0068 (5)
O7	0.0439 (7)	0.0989 (10)	0.0628 (7)	−0.0173 (6)	−0.0012 (6)	−0.0441 (7)
O10	0.0351 (5)	0.0303 (5)	0.0485 (6)	−0.0063 (4)	0.0100 (4)	−0.0159 (4)
C20	0.0484 (9)	0.0480 (9)	0.0520 (9)	−0.0139 (7)	0.0082 (8)	−0.0130 (7)
C21	0.0389 (8)	0.0382 (8)	0.0414 (8)	−0.0117 (6)	0.0102 (7)	−0.0181 (6)
C22	0.0516 (9)	0.0422 (9)	0.0537 (9)	−0.0166 (7)	0.0031 (8)	−0.0189 (7)
C23	0.0552 (10)	0.0590 (11)	0.0692 (11)	−0.0273 (9)	0.0082 (9)	−0.0298 (9)
C24	0.0468 (10)	0.0835 (14)	0.0627 (11)	−0.0240 (10)	0.0060 (9)	−0.0373 (10)
C25	0.0486 (10)	0.0716 (12)	0.0551 (10)	−0.0090 (9)	−0.0020 (8)	−0.0146 (9)
C26	0.0404 (8)	0.0322 (7)	0.0480 (9)	−0.0109 (6)	0.0092 (7)	−0.0180 (6)
O27	0.0577 (7)	0.0345 (6)	0.0870 (8)	−0.0044 (5)	−0.0161 (7)	−0.0205 (6)
O28	0.0441 (6)	0.0321 (5)	0.0852 (8)	−0.0119 (5)	−0.0046 (6)	−0.0178 (5)

### Geometric parameters (Å, º)

**Table:**

C1—C2	1.377 (2)	C14—C15	1.5229 (19)
C1—C6	1.389 (2)	C15—H15	0.9800
C1—O7	1.3739 (19)	C15—C16	1.5204 (18)
C2—H2	0.9300	C16—H16A	0.9700
C2—C3	1.379 (2)	C16—H16B	0.9700
C3—H3	0.9300	C16—N17	1.5000 (17)
C3—C4	1.381 (2)	C18—H18A	0.9600
C4—H4	0.9300	C18—H18B	0.9600
C4—C5	1.3881 (18)	C18—H18C	0.9600
C5—C6	1.3944 (19)	C18—N17	1.4771 (17)
C5—C9	1.5359 (19)	C19—H19A	0.9600
C6—H6	0.9300	C19—H19B	0.9600
C8—H8A	0.9600	C19—H19C	0.9600
C8—H8B	0.9600	C19—N17	1.4833 (18)
C8—H8C	0.9600	N17—H17	0.9800
C8—O7	1.4126 (19)	O10—H10	0.8200
C9—C11	1.5314 (19)	C20—H20	0.9300
C9—C15	1.5507 (18)	C20—C21	1.385 (2)
C9—O10	1.4257 (15)	C20—C25	1.386 (2)
C11—H11A	0.9700	C21—C22	1.389 (2)
C11—H11B	0.9700	C21—C26	1.502 (2)
C11—C12	1.515 (2)	C22—H22	0.9300
C12—H12A	0.9700	C22—C23	1.376 (2)
C12—H12B	0.9700	C23—H23	0.9300
C12—C13	1.522 (2)	C23—C24	1.376 (2)
C13—H13A	0.9700	C24—H24	0.9300
C13—H13B	0.9700	C24—C25	1.379 (2)
C13—C14	1.521 (2)	C25—H25	0.9300
C14—H14A	0.9700	C26—O27	1.2412 (17)
C14—H14B	0.9700	C26—O28	1.2661 (17)
			
C2—C1—C6	120.59 (14)	C9—C15—H15	107.9
O7—C1—C2	115.75 (14)	C14—C15—C9	110.98 (11)
O7—C1—C6	123.65 (14)	C14—C15—H15	107.9
C1—C2—H2	120.2	C16—C15—C9	109.26 (10)
C1—C2—C3	119.53 (14)	C16—C15—C14	112.67 (11)
C3—C2—H2	120.2	C16—C15—H15	107.9
C2—C3—H3	119.9	C15—C16—H16A	108.4
C2—C3—C4	120.17 (15)	C15—C16—H16B	108.4
C4—C3—H3	119.9	H16A—C16—H16B	107.5
C3—C4—H4	119.4	N17—C16—C15	115.49 (10)
C3—C4—C5	121.20 (14)	N17—C16—H16A	108.4
C5—C4—H4	119.4	N17—C16—H16B	108.4
C4—C5—C6	118.22 (13)	H18A—C18—H18B	109.5
C4—C5—C9	121.36 (12)	H18A—C18—H18C	109.5
C6—C5—C9	120.36 (12)	H18B—C18—H18C	109.5
C1—C6—C5	120.28 (13)	N17—C18—H18A	109.5
C1—C6—H6	119.9	N17—C18—H18B	109.5
C5—C6—H6	119.9	N17—C18—H18C	109.5
H8A—C8—H8B	109.5	H19A—C19—H19B	109.5
H8A—C8—H8C	109.5	H19A—C19—H19C	109.5
H8B—C8—H8C	109.5	H19B—C19—H19C	109.5
O7—C8—H8A	109.5	N17—C19—H19A	109.5
O7—C8—H8B	109.5	N17—C19—H19B	109.5
O7—C8—H8C	109.5	N17—C19—H19C	109.5
C5—C9—C15	111.34 (10)	C16—N17—H17	107.6
C11—C9—C5	107.75 (10)	C18—N17—C16	112.99 (11)
C11—C9—C15	110.47 (11)	C18—N17—C19	110.77 (12)
O10—C9—C5	110.85 (11)	C18—N17—H17	107.6
O10—C9—C11	111.47 (11)	C19—N17—C16	110.13 (10)
O10—C9—C15	105.01 (10)	C19—N17—H17	107.6
C9—C11—H11A	108.6	C1—O7—C8	117.87 (12)
C9—C11—H11B	108.6	C9—O10—H10	109.5
H11A—C11—H11B	107.6	C21—C20—H20	119.7
C12—C11—C9	114.67 (12)	C21—C20—C25	120.58 (16)
C12—C11—H11A	108.6	C25—C20—H20	119.7
C12—C11—H11B	108.6	C20—C21—C22	118.47 (14)
C11—C12—H12A	109.6	C20—C21—C26	120.42 (13)
C11—C12—H12B	109.6	C22—C21—C26	121.10 (13)
C11—C12—C13	110.42 (12)	C21—C22—H22	119.5
H12A—C12—H12B	108.1	C23—C22—C21	120.94 (15)
C13—C12—H12A	109.6	C23—C22—H22	119.5
C13—C12—H12B	109.6	C22—C23—H23	119.9
C12—C13—H13A	109.6	C24—C23—C22	120.10 (16)
C12—C13—H13B	109.6	C24—C23—H23	119.9
H13A—C13—H13B	108.1	C23—C24—H24	120.1
C14—C13—C12	110.39 (12)	C23—C24—C25	119.87 (16)
C14—C13—H13A	109.6	C25—C24—H24	120.1
C14—C13—H13B	109.6	C20—C25—H25	120.0
C13—C14—H14A	109.3	C24—C25—C20	120.03 (16)
C13—C14—H14B	109.3	C24—C25—H25	120.0
C13—C14—C15	111.70 (12)	O27—C26—C21	120.10 (13)
H14A—C14—H14B	107.9	O27—C26—O28	124.15 (14)
C15—C14—H14A	109.3	O28—C26—C21	115.75 (13)
C15—C14—H14B	109.3		
			
C1—C2—C3—C4	−0.4 (2)	C12—C13—C14—C15	59.28 (17)
C2—C1—C6—C5	1.2 (2)	C13—C14—C15—C9	−56.84 (15)
C2—C1—O7—C8	174.83 (15)	C13—C14—C15—C16	−179.74 (12)
C2—C3—C4—C5	0.4 (2)	C14—C15—C16—N17	−63.19 (16)
C3—C4—C5—C6	0.4 (2)	C15—C9—C11—C12	−51.00 (15)
C3—C4—C5—C9	−176.79 (13)	C15—C16—N17—C18	−59.79 (16)
C4—C5—C6—C1	−1.23 (19)	C15—C16—N17—C19	175.76 (12)
C4—C5—C9—C11	71.47 (15)	O7—C1—C2—C3	178.80 (14)
C4—C5—C9—C15	−49.81 (16)	O7—C1—C6—C5	−177.88 (13)
C4—C5—C9—O10	−166.32 (11)	O10—C9—C11—C12	65.34 (15)
C5—C9—C11—C12	−172.82 (11)	O10—C9—C15—C14	−68.97 (13)
C5—C9—C15—C14	171.00 (10)	O10—C9—C15—C16	55.87 (13)
C5—C9—C15—C16	−64.15 (13)	C20—C21—C22—C23	0.7 (2)
C6—C1—C2—C3	−0.3 (2)	C20—C21—C26—O27	164.70 (14)
C6—C1—O7—C8	−6.0 (2)	C20—C21—C26—O28	−16.30 (19)
C6—C5—C9—C11	−105.71 (14)	C21—C20—C25—C24	−1.2 (2)
C6—C5—C9—C15	133.01 (13)	C21—C22—C23—C24	−1.4 (2)
C6—C5—C9—O10	16.51 (16)	C22—C21—C26—O27	−16.0 (2)
C9—C5—C6—C1	176.03 (12)	C22—C21—C26—O28	163.00 (13)
C9—C11—C12—C13	53.62 (16)	C22—C23—C24—C25	0.8 (2)
C9—C15—C16—N17	172.96 (11)	C23—C24—C25—C20	0.5 (2)
C11—C9—C15—C14	51.32 (14)	C25—C20—C21—C22	0.6 (2)
C11—C9—C15—C16	176.17 (11)	C25—C20—C21—C26	179.94 (13)
C11—C12—C13—C14	−56.17 (17)	C26—C21—C22—C23	−178.64 (13)

### Hydrogen-bond geometry (Å, º)

Cg1 is the centroid of the C1–C6 benzene ring.

**Table:**

*D*—H···*A*	*D*—H	H···*A*	*D*···*A*	*D*—H···*A*
C6—H6···O10	0.93	2.42	2.775 (2)	102
C14—H14*B*···O28^i^	0.97	2.41	3.251 (2)	144
N17—H17···O28^i^	0.98	1.65	2.6120 (18)	165
O10—H10···O27^ii^	0.82	1.94	2.7269 (17)	161
C23—H23···*Cg*1^iii^	0.93	2.83	3.684 (3)	153

Symmetry codes: (i) *x*+1, *y*, *z*; (ii) *x*+1, *y*−1, *z*; (iii) *x*, *y*+1, *z*.
