# Supplementary figures and images for: Crystal structure of {[2-hy­droxy-2-(3-meth­oxy­phen­yl)cyclo­hex­yl]meth­yl}di­methyl­ammonium benzoate
Source: Acta Crystallogr E Crystallogr Commun. 2015 Oct 17;71(Pt 11):o864–5. doi: 10.1107/S2056989015019362 (PMC4645060; doi:10.1107/S2056989015019362)

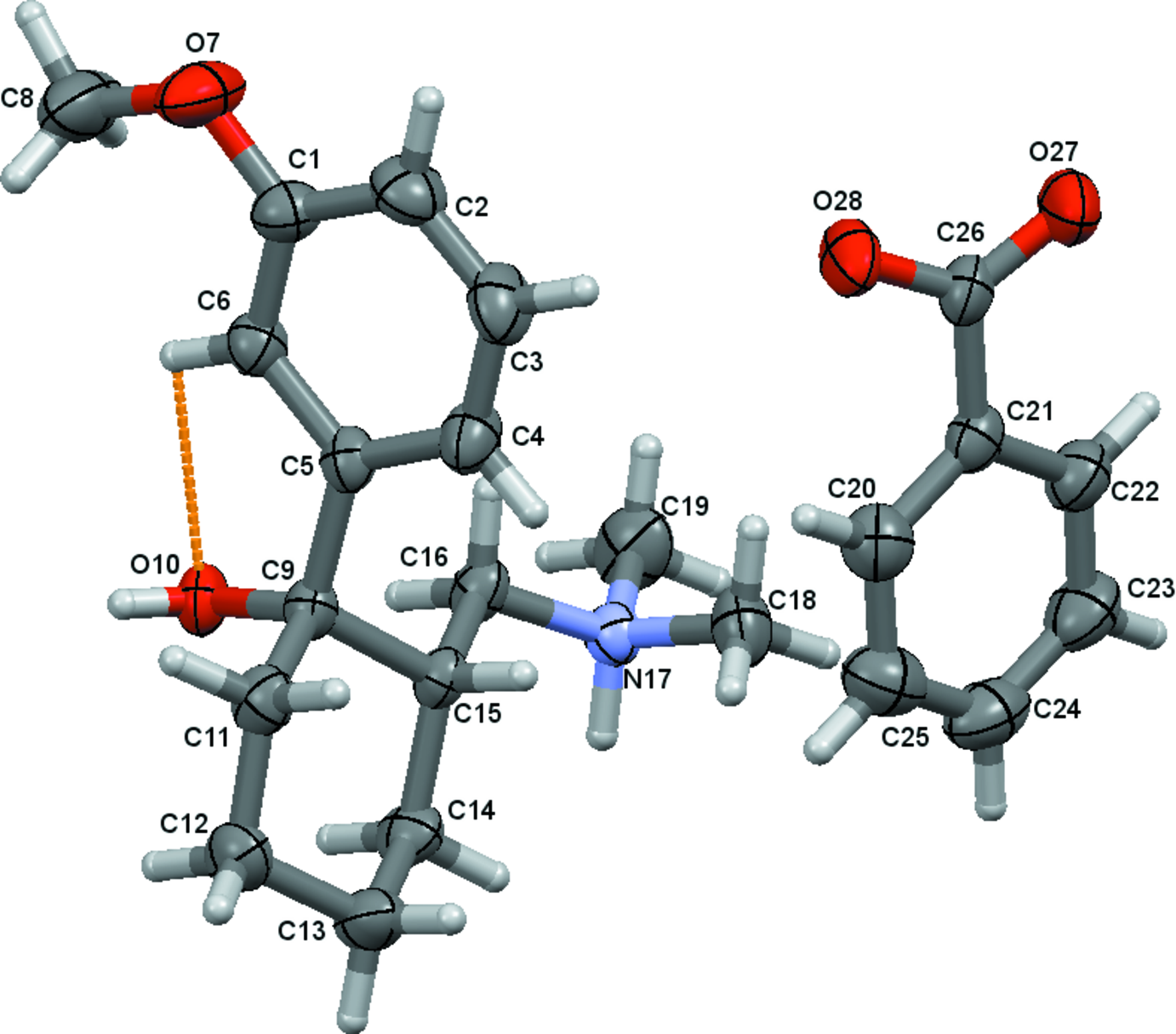

Supplement: Supplementary file 4 [file e-71-0o864-fig1.tif]

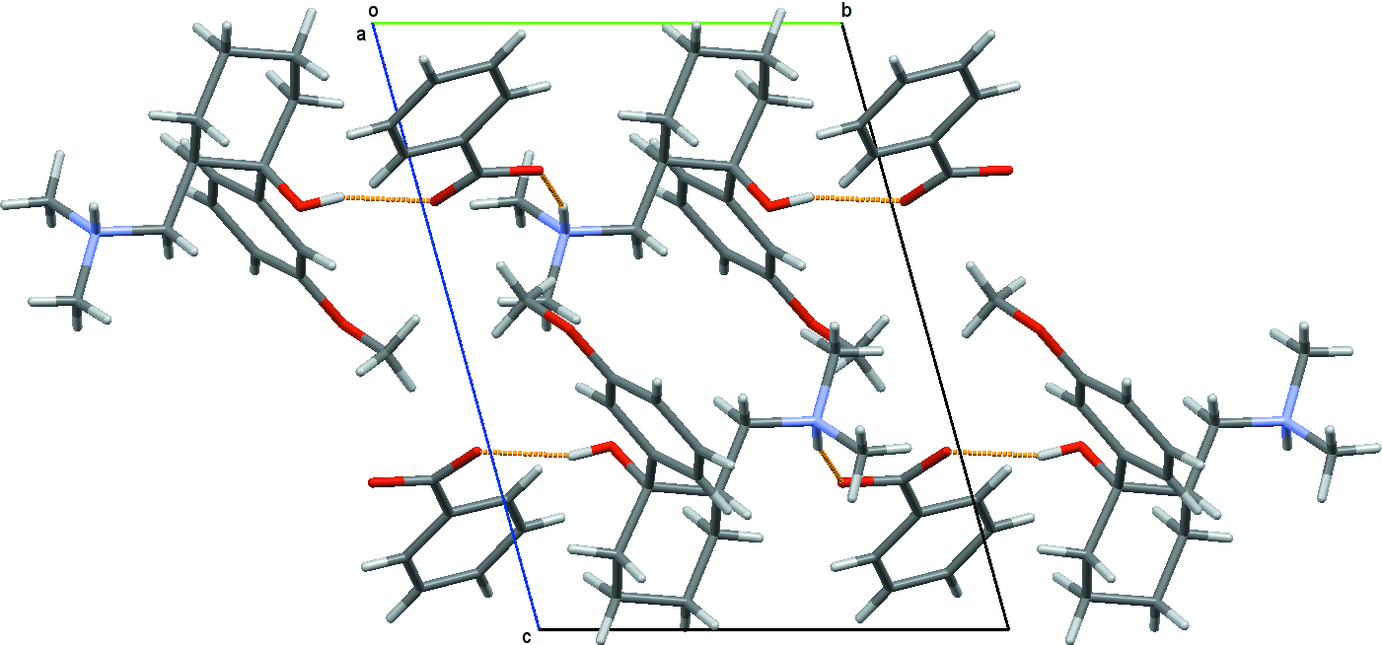

Supplement: Supplementary file 5 [file e-71-0o864-fig2.tif]
